# Low total cholesterol level is the independent predictor of poor outcomes in patients with acute ischemic stroke: a hospital-based prospective study

**DOI:** 10.1186/s12883-016-0561-z

**Published:** 2016-03-15

**Authors:** Wenjuan Zhao, Zhongping An, Yan Hong, Guanen Zhou, Jingjing Guo, Yongli Zhang, Yuanju Yang, Xianjia Ning, Jinghua Wang

**Affiliations:** Department of Neurology, Tianjin Huanhu Hospital, 122 Qixiangtai Road, Hexi District, Tianjin, 300060 China; Tianjin Key Laboratory of Cerebral Vascular and Neurodegenerative Disease, Tianjin, China; Department of Radiology, Tianjin Huanhu Hospital, Tianjin, China; Department of Clinical Pharmacy, Tianjin Huanhu Hospital, Tianjin, China; Department of Epidemiology, Tianjin Neurological Institute & Department of Neurology, Tianjin Medical University General Hospital, Tianjin, China

**Keywords:** Total cholesterol, Atherothrombotic ischemic stroke, Outcomes

## Abstract

**Background:**

Total cholesterol is a well-documented risk factor for coronary disease. Previous studies have shown that high total cholesterol level is associated with better stroke outcomes, but the association of low total cholesterol levels and ischemic stroke outcomes is rare. Therefore, we aimed to assess the association of low total cholesterol levels and stroke outcomes among acute ischemic stroke patients in China.

**Methods:**

This study recruited 6407 atherothrombotic infarction patients from Tianjin, China, between May 2005 and September 2014. All patients were categorized into five groups according to TC level quintiles at admission. Differences in subtypes, severity, risk factors, and outcomes at 3, 12, and 36 months after stroke were compared between these groups.

**Results:**

In total, 1256 (19.6 %) patients had low cholesterol levels, with a higher prevalence in men than in women (23.7 % vs. 11.2 %, *P* < 0.001). Compared with higher cholesterol levels, the lowest cholesterol level quintile (TC, <4.07 mmol/L) was associated with older age (64.7 years, *P* = 0.033), anterior circulation infarct (22.8 %), atrial fibrillation (4.9 %), current smoking (41.1 %), and alcohol consumption (21.1 %) and lower frequencies of hypertension (72.9 %), diabetes (30.7 %), and obesity (9.9 %). Dependency and recurrence rates were significantly higher at 36 months in patients in the lowest TC level quintile than in those with higher cholesterol levels (dependency rates, 51.2 % vs 45.2 %; *P* = 0.007 and recurrence rates, 46.3 % vs 37.3 %, *P* = 0.001). Moreover, these differences remained after adjustment for age, sex, stroke severity, and Oxfordshire Community Stroke Project classification (odds ratios [ORs] for dependency rate, 1.41; 95 % confidence interval [CI], 1.11, 1.79; *P* = 0.005 and recurrence rate, 1.50; 95 % CI, 1.19, 1.89; *P* = 0.001). However, mortality rates after stroke were not significantly different between the groups.

**Conclusions:**

These findings suggest that statin treatment for patients with atherothrombotic infarction and low cholesterol levels increase long-term dependency and recurrence rates, but do not increase mortality rates. It is crucial to highlight the different impact of statin treatment on patients with atherothrombotic infarction and lower cholesterol levels for secondary stroke prevention in China.

## Background

In 2010, stroke was the second most common cause of death and the third most common cause of reduced disability-adjusted life-years worldwide. Although age-standardized rates of stroke mortality have decreased worldwide in the past two decades, the absolute numbers of annual stroke cases, stroke survivors, related deaths, and the global burden of stroke disability-adjusted life-years are high and are increasing [1, 2]. However, stroke has recently become the leading cause of death in rural areas, and the third most common cause of death in urban areas in China [3]. High total cholesterol (TC) level is a well-documented risk factor for coronary disease [4, 5], but the association between total cholesterol levels and stroke outcome is unclear. A large numbers of studies indicated that high TC level was associated with better stroke outcomes [6–8], but high TC level was associated with worse outcomes in other studies [9, 10]. Low TC levels were associated with hemorrhagic, but not ischemic stroke [11, 12]. The recent incidence of stroke in China has increased dramatically, with economic development [13]; however, a large-scale study of the association between TC level and stroke outcomes is rare in China, especially in patients with atherothrombotic infarction.

Therefore, we aimed to assess the association of low TC level on admission and short-term and long-term stroke outcomes after acute ischemic stroke (AIS) in patients in China.

## Methods

### Subjects

All consecutive patients with first-ever AIS who were admitted to the Stroke Unit in Tianjin Huanhu Hospital within 72 h of stroke onset between in May 2005 and September 2014 were recruited to this study. A clinical diagnosis of stroke was made according to the World Health Organization criteria and confirmed by neuroimaging (computed tomography/magnetic resonance imaging) [14]. Cases of transient ischemic attack were excluded from this study, and all atherothrombotic infarction patients classified according to the Trial of Org 10,172 in Acute Stroke Treatment (TOAST) for large artery atherothrombotic and small artery occlusion (SAO) were analyzed in this study [15]. All patients were treated using statin, and followed up for less than 3 months after AIS and TC level on admission was available.

The study was approved by the ethics committee for medical research at Tianjin Huanhu Hospital and the Tianjin Health Bureau, and a written informed consent for each participant was obtained during recruitment.

### Data collection and group

The detailed information on ischemic stroke subtype, stroke severity, previous history of diseases, stroke risk factors, laboratory examination results, and outcomes at 3, 12, and 36 months after stroke were collected using a standardized questionnaire.

All patients were categorized into five groups according to the TC level quintile at admission: group 1, <4.07 mmol/L; group 2, 4.07–4.61 mmol/L; group 3, 4.62–5.15 mmol/L; group 4, 5.16–5.81 mmol/L; and group 5, >5.81 mmol/L.

### Stroke subtypes

Stroke subtypes were defined as total anterior circulation infarct (TACI), partial anterior circulation infarct (PACI), posterior circulation infarct (POCI), and lacunar infarct (LACI) according to Oxfordshire Community Stroke Project (OCSP) classification criteria [16].

### Neurological function deficit and stroke severity

Neurological function deficit was defined using the National Institute of Health stroke scale (NIHSS), Barthel index (BI) [17], and modified rankin scale (mRS) on admission [18]. Stroke severity was categorized into 3 groups on the basis of NIHSS score: mild (NIHSS ≤7), moderate (NIHSS between 8 to 16), and severe (NIHSS ≥ 17) [19].

### Risk factors

Stroke risk factors included a medical history of hypertension (defined as self-reported history of hypertension or using antihypertension drugs), diabetes mellitus (DM, defined as history of DM or using hypoglycemic medications at discharge), atrial fibrillation (AF, defined as history of AF, confirmed by at least one electrocardiogram or the presence of the arrhythmia during hospitalization); and modifiable lifestyle factors, including current smoking status, alcohol consumption, and obesity (body mass index ≥30 kg/m^2^).

### Outcome definition

Stroke outcomes were described on the basis of mortality, recurrence, and dependency rates at the short-term (at 3 months), medium-term (at 12 months), and long-term (at 36 months) after stroke; outcomes were assessed using face-to-face or telephone follow-up. Death was defined as all-cause cumulative death at the corresponding time points after stroke, and this information was collected from patients’ family members by telephone follow-up. Recurrence was defined as new-onset vascular events (stroke, myocardial infarction, and venous thrombosis) after 30 days of initial stroke in all survivors patients who were followed up using face-to race interviews or telephone calls. Dependency rate was defined as mRS > 2 among all survivors followed up using face-to race interview or telephone calls [20].

### Follow-up periods

Follow-up was conducted according to a predetermined procedure; the trained neurologists reexamined patients in the outpatient department at 3, 12, 24, and 36 months after stroke. All patients were followed-up by face-to-face interview, but those patients reexamined in the local hospital were followed-up by telephone.

### Statistical analysis

Age is presented as mean (standard deviation), and NIHSS, BI, and mRS are presented as median (interquartile range). These continuous variables were compared between the five TC groups using one-way analysis of variance (ANOVA) or Kruskal-Wallis tests. At the different time periods after stroke, categorical variables, including stroke subtype, stroke severity, risk factors, and outcomes, are presented as number (percentage), and the trends were compared using Chi-square tests. Differences in outcomes were compared between the five groups using univariate and multivariate logistic regression models and are presented as unadjusted and adjusted (by sex, age, stroke severity, OCSP, and risk factors [hypertension, diabetes, atrial fibrillation, artery stenosis, obesity, current smoking, and alcohol consumption]) odds ratios (OR), respectively, with 95 % confidence intervals (CI). All statistical analyses were performed using SPSS version 15.0 (SPSS Inc., Chicago, IL), and two-tailed *P* values <0.05 were considered statistically significant.

## Results

**Fig. 1:**
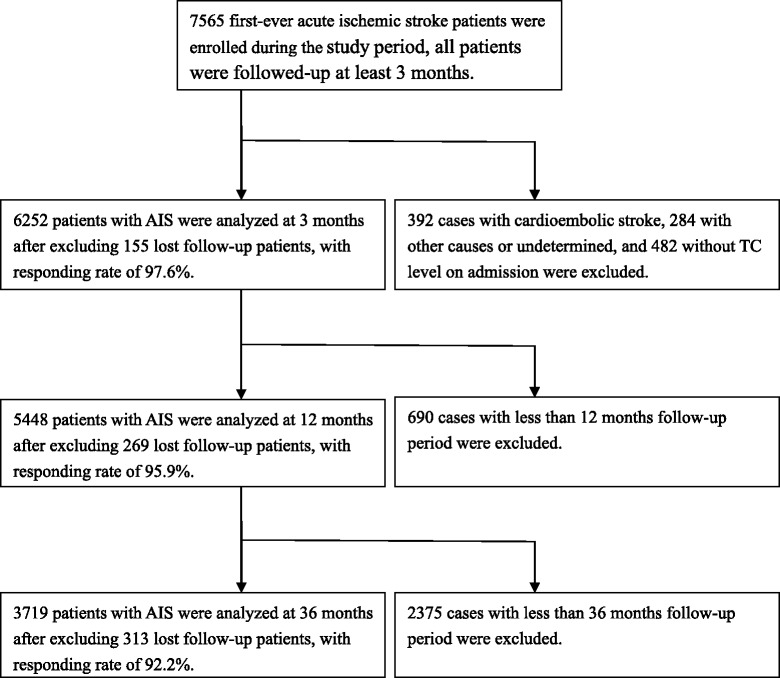
Flow diagram of participants

Of the 7565 AIS patients recruited between May 2005 and September 2014, 392 patients with cardioembolic stroke, 284 patients with other and undetermined causes and 482 patients without a TC level recorded at admission were excluded, resulting in 6407 patients with atherothrombotic infarction that were included. At 3 months, 155 patients were lost to follow-up, resulting in 6252 patients (response rate, 97.6 %). At 12 months, 269 patients were lost to follow-up, resulting in 5448 patients (response rate, 95.3 %). At 36 months, 313 patients were lost to follow-up, resulting in 3719 patients (response rate, 92.2 %) (Fig. 1).

The lowest TC levels were present in 1256 (19.6 %) patients (1022 [23.7 %] men; 234 [11.2 %] women; *P* < 0.001; Table 1). The mean age at stroke onset decreased with increasing TC levels (*P* = 0.033). The prevalence of TACI decreased, and the prevalence of POCI increased, with increasing TC levels (*P* < 0.001). Moreover, the proportion of patients with severe stroke increased with increasing TC levels (*P* < 0.05); neurological function deficits at admission were worse with increasing TC levels (*P* < 0.05).

**Table 1:** The clinical characteristics and risk factors in patients with acute ischemic stroke by TC levels

Characteristics	Group 1	Group 2	Group 3	Group 4	Group 5	*P*
Numbers, *n* (%)	1256 (19.6)	1282 (20.0)	1283 (20.0)	1312 (20.5)	1274 (19.9)	—
Gender, *n* (%)						<0.001
Men	1022 (23.7)	959 (22.2)	907 (21.0)	794 (18.4)	637 (14.7)	
Women	234 (11.2)	323 (15.5)	376 (18.0)	518 (24.8)	637 (30.5)	
Age, year, means (SD)	64.69 (11.55)	64.10 (11.46)	63.59 (11.43)	63.36 (10.98)	63.94 (11.12)	0.033
OCSP classification, *n* (%):						<0.001
TACI	63 (22.8)	59 (21.4)	54 (19.6)	46 (16.7)	54 (19.6)	
PACI	720 (20.7)	688 (19.8)	704 (20.2)	716 (20.6)	651 (18.7)	
LACI	82 (18.2)	118 (26.2)	85 (18.8)	90 (20.0)	76 (16.9)	
POCI	391 (17.8)	417 (18.9)	440 (20.0)	460 (20.9)	493 (22.4)	
Stroke severity:						0.001
Mild	849 (67.6)	907 (70.7)	886 (69.1)	889 (67.8)	803 (63.0)	
Moderate	309 (24.6)	282 (22.0)	311 (24.2)	320 (24.4)	337 (26.5)	
Severe	98 (7.8)	93 (7.3)	86 (6.7)	102 (7.8)	134 (10.5)	
Neurological function deficit:						
NIHSS	5 (7)	5 (6)	5 (7)	5 (7)	5 (7)	0.022
BI	60 (45)	60 (45)	60 (45)	60 (45)	60 (55)	0.006
mRS	3 (2)	3 (2)	3 (2)	3 (2)	3 (2)	0.006

*TC* total cholesterol, *OCSP* Oxfordshire Community Stroke Project, *TACI* total anterior circulation infarct, *PACI* partial anterior circulation infarct, *POCI* posterior circulation infarct, *LACI* lacunar infarct, *NIHSS* National Institute of Health stroke scale, *BI* Barthel index, *mRS* modified rankin scale

The prevalence of AF, current smoking, and alcohol consumption were significantly lower with higher TC levels (all *P* < 0.05). In contrast, the prevalence of hypertension, diabetes, and obesity increased with increasing TC levels (all *P* < 0.001). The prevalence of artery stenosis was not significantly different (Table 2).

**Table 2:** The Prevalence of risk factors in patients with acute ischemic stroke by TC levels

Risk factors	Total	Group 1	Group 2	Group 3	Group 4	Group 5	*P*
Hypertension	4756 (74.2)	916 (72.9)	922 (71.9)	933 (72.7)	987 (75.2)	998 (78.3)	<0.001
Diabetes	2090 (32.6)	386 (30.7)	370 (28.9)	418 (32.6)	411 (31.3)	505 (39.6)	<0.001
Atrial fibrillation	231 (3.6)	62 (4.9)	46 (3.6)	41 (3.2)	46 (3.5)	36 (2.8)	0.010
Artery stenosis	1572 (24.5)	339 (27.0)	297 (23.2)	295 (23.0)	317 (24.2)	324 (25.4)	0.589
Obesity	795 (12.4)	124 (9.9)	141 (11.0)	130 (10.1)	192 (14.6)	208 (16.3)	<0.001
Current smoking	2565 (40.0)	516 (41.1)	575 (44.9)	524 (40.8)	511 (38.9)	439 (34.5)	<0.001
Alcohol consumption	1243 (19.4)	265 (21.1)	252 (19.7)	253 (19.7)	247 (18.8)	226 (17.7)	0.032

*TC* total cholesterol

There were no obvious differences in mortality and dependency rates at all time points after AIS between the TC level groups (Table 3). However, the recurrence rate at 3 months was remarkably higher with higher TC levels (group 1, 7.0 %; group 2, 7.4 %; group 3, 9.8 %; group 4, 9.7 %; and group 5, 8.6 %; *P* = 0.038). The trend in recurrence rates at 36 months after stroke was the opposite to that at 3 months (group 1, 46.3 %; group 2, 41.0 %; group 3, 41.1 %; group 4, 37.7 %; and group 5, 37.3 %; *P* = 0.001).

**Table 3:** The outcome at 3, 12, and 36 months after stroke in acute ischemic stroke patients by TC levels

Outcomes	Total	Group 1	Group 2	Group 3	Group 4	Group 5	*P*
3 months:							
Mortality	340 (5.4)	76 (6.2)	57 (4.6)	54 (4.3)	73 (5.7)	80 (6.5)	0.414
Dependency	602 (10.1)	124 (10.7)	125 (10.3)	108 (8.9)	111 (9.1)	134 (11.4)	0.962
Recurrence	207 (8.5)	81 (7.0)	90 (7.4)	118 (9.8)	118 (9.7)	100 (8.6)	0.038
12 months:							
Mortality	454 (8.3)	91 (8.6)	87 (7.9)	68 (6.3)	94 (8.4)	114 (10.6)	0.080
Dependency	1218 (24.4)	258 (26.6)	239 (23.4)	234 (22.9)	250 (24.4)	237 (24.7)	0.538
Recurrence	1153 (22.7)	229 (23.3)	219 (20.9)	261 (25.4)	229 (22.0)	215 (21.8)	0.647
36 months:							
Mortality	588 (15.8)	109 (15.5)	127 (16.1)	103 (13.6)	117 (15.9)	132 (18.2)	0.229
Dependency	1447 (46.2)	305 (51.2)	301 (45.4)	290 (44.1)	282 (45.5)	269 (45.2)	0.070
Recurrence	1359 (40.7)	294 (46.3)	291 (41.0)	289 (41.1)	249 (37.7)	236 (37.3)	0.001

*TC* total cholesterol, *OR* odds ratios, *CI* confidence intervals

In the univariate analysis, compared with the lowest TC levels (group 1), mortality rates were lower at 3 and 12 months after stroke (by 32 and 29 %, respectively), and the recurrence rate was lower at 36 months after stroke (by 20 %) in those with TC levels of 4.62–5.15 mmol/L (group 3). Compared with the lowest TC level group (group 1), the dependency rate was 21 % lower in group 2, 25 % lower in group 3, 20 % lower in group 4, and 21 % lower in group 5 (Table 4).

**Table 4:** Un-adjusted OR (95 % CI) of TC levels in the outcomes at 3, 12, and 36 months after stroke in acute ischemic stroke patients

Outcomes	Group 1	Group 2	Group 3	Group 4	Group 5
3 months:					
Mortality	1.00	0.72 (0.51, 1.03)	0.68 (0.47, 0.97)^a^	0.91 (0.66, 1.27)	1.04 (0.75, 1.44)
Dependency	1.00	0.96 (0.74, 1.24)	0.81 (0.62, 1.07)	0.83 (0.64, 1.09)	1.07 (0.83, 1.39)
Recurrence	1.00	0.87 (0.66, 1.14)	0.80 (0.60, 1.05)	0.79 (0.60, 1.05)	1.02 (0.78, 1.33)
12 months:					
Mortality	1.00	0.91 (0.67, 1.23)	0.71 (0.51, 0.99)^a^	0.98 (0.72, 1.32)	1.27 (0.95, 1.70)
Dependency	1.00	0.84 (0.69, 1.03)	0.82 (0.67, 1.01)	0.89 (0.73, 1.09)	0.91 (0.74, 1.11)
Recurrence	1.00	0.90 (0.74, 1.09)	0.85 (0.70, 1.04)	0.93 (0.76, 1.13)	0.94 (0.77, 1.15)
36 months:					
Mortality	1.00	1.05 (0.79, 1.38)	0.86 (0.64, 1.15)	1.03 (0.78, 1.37)	1.21 (0.92, 1.60)
Dependency	1.00	0.79 (0.64, 0.99)^a^	0.75 (0.60, 0.94)^a^	0.80 (0.64, 0.99)^a^	0.79 (0.63, 0.99)^a^
Recurrence	1.00	0.87 (0.70, 1.07)	0.80 (0.64, 0.99)^a^	0.83 (0.67, 1.03)	0.82 (0.66, 1.01)

*TC* total cholesterol, *OR* odds ratios, *CI* confidence intervals

^a^ indicated P<0.05 refence as Group 1

In the multivariate analysis, the dependency rate was significantly lower in the higher TC level groups compared with group 1, by 21 % in group 2, 24 % in group 3, 22 % in group 4, and 29 % in group 5. The recurrence rates in TC level groups 3 and 5 were significantly lower (by 20 and 27 %, respectively) than that in the lowest TC group (group 1; Table 5).

**Table 5:** Adjusted^a^ OR (95 % CI) of TC levels in the outcomes at 3, 12, and 36 months after stroke in acute ischemic stroke patients

Outcomes	Group 1	Group 2	Group 3	Group 4	Group 5
3 months:					
Mortality	1.00	0.77 (0.52, 1.13)	0.73 (0.49, 1.08)	1.08 (0.74, 1.55)	0.99 (0.68, 1.44)
Dependency	1.00	0.98 (0.75, 1.29)	0.84 (0.63, 1.10)	0.88 (0.67, 1.17)	1.04 (0.79, 1.37)
Recurrence	1.00	0.89 (0.68, 1.18)	0.82 (0.62, 1.09)	0.85 (0.64, 1.13)	1.02 (0.77, 1.35)
12 months:					
Mortality	1.00	0.99 (0.70, 1.39)	0.74 (0.52, 1.07)	1.11 (0.79, 1.57)	1.22 (0.87, 1.71)
Dependency	1.00	0.86 (0.70, 1.06)	0.83 (0.68, 1.03)	0.92 (0.75, 1.13)	0.87 (0.71, 1.09)
Recurrence	1.00	0.91 (0.74, 1.11)	0.86 (0.70, 1.05)	0.95 (0.77, 1.16)	0.89 (0.72, 1.10)
36 months:					
Mortality	1.00	1.15 (0.84, 1.58)	0.88 (0.63, 1.23)	1.12 (0.81, 1.56)	1.07 (0.77, 1.49)
Dependency	1.00	0.79 (0.63, 0.99)^a^	0.76 (0.60, 0.95)^a^	0.78 (0.62, 0.99)^a^	0.71 (0.56, 0.90)^a^
Recurrence	1.00	0.86 (0.70, 1.07)	0.80 (0.64, 0.99)^a^	0.81 (0.64, 1.01)	0.73 (0.58, 0.91)^a^

*TC* total cholesterol, *OR* odds ratios, *CI* confidence intervals

^a^Adjusted by sex, age, stroke severity, OCSP, risk factors (hypertension, diabetes, atrial fibrillation, artery stenosis, obesity, current smoking, and alcohol consumption) as covariates

## Discussion

In this single-center study using a large stroke registry in Tianjin, China, we assessed differences in age, sex, stroke subtype, stroke severity, prevalence of risk factors, and stroke outcomes between patients with atherothrombotic infarction with and without low TC levels. As a result, a low TC level was an independent risk factor for outcomes in patients with atherothrombotic infarction.

A high cholesterol level is a powerful risk factor for coronary heart disease, but its role in stroke remains controversial. Although observational studies have not found a clear association between cholesterol levels and stroke [21–25], associations between high serum TC levels and an increased risk of ischemic stroke have been reported [26, 27]. A study in Americans indicated a positive association of TC levels with atherothrombotic infarction [28]. Only one study conducted in Japan reported that serum TC levels were positively associated with an increased age-adjusted risk of atherothrombotic infarction in Japanese women aged ≥40 years, but not in men [29].

A recent study showed that serum cholesterol <160 mg/dL was a risk factor for hemorrhagic stroke and increased the risk of death after hemorrhagic stroke, but a reverse trend was found for ischemic stroke incidence and death [11, 12]. Moreover, a study conducted in Japan indicated that a higher TC level increased the risk of cerebral infarction [11]. In a hospital-based study, low serum TC levels were associated with increased risk of severe stroke, TACI, and poor functional outcomes in patients with ischemic stroke who had received pre-stroke statin treatment, and the short-term and long-term mortality rates were significantly higher in patients with low cholesterol levels [30]. Another community-based cohort study indicated that low cholesterol levels significantly increased the risks of stroke and heart disease [31]. In contrast with these previous findings, there was no significant association between low cholesterol levels and mortality after stroke in the present study.

A meta-analysis including 450,000 individuals from 45 observational cohorts suggested that there was no association between TC level and the risk of fatal stroke during an average follow-up of 16 years [32]. In another meta-analysis of approximately 1 million individuals, a clear association was found between the serum TC level and the risk of fatal myocardial infarction, but again, there was no obvious association with fatal stroke [25].

Consistent with the findings of previous studies, the present study found that, compared with patients with higher TC levels, patients with low TC levels were more likely to be male; older; to have TACI or AF; be a current smoker; and currently consume alcohol. Regarding age, the TC level was associated with fatal ischemic stroke in patients aged <70 years, but not in patients aged >70 years in a previous study [25]. Moreover, patients in the lowest TC level group were less likely to have hypertension, diabetes, and obesity than patients in the higher TC level groups.

Clinical trials using statins to reduce cholesterol levels in patients with cardiovascular or cerebrovascular diseases have shown significant reductions in the risk of stroke [33–39] and that reduced cholesterol levels can reduce the incidence of stroke in high-risk populations and in patients with a stroke or transient ischemic attack [25, 32]. Statin therapy has become the most important advancement in stroke prevention since aspirin and blood pressure-lowering therapies were introduced. Statins not only lower the overall risk of stroke but also reduce the progression of carotid atherosclerosis; reduce inflammation and endothelial dysfunction; decrease platelet aggregation to improve fibrinolysis; lower blood pressure; and decrease the risk of thromboembolic complications in the brain by reducing the incidence of myocardial infarction. Statins might also have a neuroprotective effect [40–42]. However, clinicians should explore the effects of statin treatment in patients with atherothrombotic infarction and low TC levels.

Given the higher dependency and recurrence rates at 36 months after stroke in patients with the lowest TC levels at admission in the present study, the benefits of statin treatment for atherothrombotic infarction did not appear to occur, supporting the role of low TC levels as an independent risk factor for outcomes in these patients. The mechanism explaining the association between low TC levels and poorer stroke outcomes is unknown. However, differences in race and social development between Asian and Western populations might partially explain the relationship.

There are several limitations in this study. First, all patients were from a local neurological hospital in Tianjin, China, and may not represent all stroke patients in China. Second, the data on TC levels before stroke was lacking; this may have affected the evaluation of statin treatment.

## Conclusion

In this large, hospital-based, prospective study using a stroke registry from Tianjin, China, we assessed differences in stroke subtype, severity, risk factors, and outcomes at 3, 12, and 36 months after stroke in patients with atherothrombotic infarction based on different TC levels. Men and older patients were more likely to have low TC levels, and the frequencies of TACI, AF, current smoking, and alcohol consumption were higher in patients with low TC levels than in patients with higher TC levels. Furthermore, low TC levels were associated with poor long-term outcomes, and a significant negative relationship between cholesterol levels and dependency and recurrence rates at 36 months after stroke onset were observed. However, there was no significant difference in mortality after stroke. Therefore, the TC level appears to be a determinant of long-term outcomes in patients with atherothrombotic infarction; statin treatment in the patients with lower TC levels increased the long-term risk of dependency and recurrence, but not the risk of all-cause death. Therefore, to improve secondary stroke prevention in China, it is important to determine the different impact of statin treatment in patients with atherothrombotic infarction based on cholesterol levels.

## Abbreviations

AF: atrial fibrillation
AIS: acute ischemic stroke
BI: bethel index
CI: confidence intervals
DM: diabetes mellitus
FG: fasting glucose
HbA1c: glycosylated hemoglobin
HDL-C: high-density lipoprotein cholesterol
LACI: lacunar infarct
LDL-C: low-density lipoprotein cholesterol
mRS: modified rank scale
NIHSS: National Institute of Health stroke scale
OCSP: Oxfordshire Community Stroke Project
PACI: partial anterior circulation infarct
POCI: posterior circulation infarct
RR: relative risk
SAO: small artery occlusion
TACI: total anterior circulation infarct
TC: total cholesterol
TG: triglyceride
TOAST: Trial of Org 10172 in Acute Stroke Treatment
